# Interleukin-17a as a predictor of occurrence of sepsis in polytrauma patients: a prospective observational study

**DOI:** 10.1186/2197-425X-3-S1-A790

**Published:** 2015-10-01

**Authors:** A Abdelkader, MAA El-Sayed, A Eladawy, R Elsayed, A Mukhtar, W Hammimy

**Affiliations:** Cairo University, Cairo, Egypt; Cairo University, Anesthesia, Cairo Egypt

## Introduction

One of the most serious complications of major trauma is the sequential dysfunction of vital organs, mostly associated with posttraumatic sepsis. IL-17 has been linked to the severity of inflammation in tissues. It initiates the production of other pro-inflammatory mediators resulting in an influx of neutrophils.

## Objectives

To investigate whether the level of IL17A at the day of admission could predict the occurrence of sepsis in polytrauma patients or not.

## Methods

A prospective cohort study in polytrauma patients. 100 consecutive adult polytrauma patients were included. Serum level of IL-17A was measured at the day of admission to the intensive care unit (ICU). Patients were screened for the development of sepsis. Other data collection included; demographic data Abbreviated Injury Scale (AIS), APACHE II score, acute kidney injury assessed by RIFLE criteria, acute respiratory distress syndrome (ARDS), duration of mechanical ventilation, ICU length of stay & 28-day mortality.

## Results

Out of 100 patients 47 (47%) developed sepsis. Serum level of IL-17A was significantly higher in the group of patients who developed sepsis compared to the non-septic group (p-value 0.004). The optimum cut-off value of serum IL-17A for sepsis prediction in polytrauma patients was ≥ 53.8 pg/ml. This cut-off value had a sensitivity of 60.7 % & a specificity of 76.4%, area under the curve (AUC) was 0.687 (95% CI 0.573-0.802), (p value 0.004), as shown in Figure 1. High serum level of IL17-A was associated with increased 28 days ICU mortality as shown in Figure 2 (p value of 0.06).

Neither ARDS nor Multiple Organ Dysfunction Score (MODS) was associated with increased IL-17A serum level.

## Conclusions

IL-17A is a potential predictor of sepsis occurrence in patients with polytrauma.
